# Molecular characterization of *carnivore protoparvovirus 1* circulating in domestic carnivores in Egypt

**DOI:** 10.3389/fvets.2022.932247

**Published:** 2022-07-22

**Authors:** Linda A. Ndiana, Gianvito Lanave, Aya A. K. Zarea, Costantina Desario, Eugene A. Odigie, Fouad A. Ehab, Paolo Capozza, Grazia Greco, Canio Buonavoglia, Nicola Decaro

**Affiliations:** ^1^Department of Veterinary Medicine, University of Bari, Bari, Italy; ^2^Department of Veterinary Microbiology, College of Veterinary Medicine, Michael Okpara University of Agriculture, Umudike, Nigeria; ^3^Department of Microbiology and Immunology, National Research Centre, Veterinary Research Institute, Giza, Egypt

**Keywords:** Carnivore protoparvovirus 1, dogs, cats, Egypt, molecular characterization, phylogeny

## Abstract

Canine parvovirus (CPV) and feline panleukopenia virus (FPV), now included in the unique species *Carnivore protoparvovirus 1* (CPPV1), have been circulating in dogs and cats for several decades and are considered the causes of clinically important diseases, especially in young animals. While genetic evidence of the circulation of parvoviruses in Egyptian domestic carnivores has been provided since 2016, to date, all available data are based on partial fragments of the VP2 gene. This study reports the molecular characterization of CPPV strains from Egypt based on the full VP2 gene. Overall, 196 blood samples were collected from dogs and cats presented at veterinary clinics for routine medical assessment in 2019 in Egypt. DNA extracts were screened and characterized by real-time PCR. Positive samples were amplified by conventional PCR and then were sequenced. Nucleotide and amino acid changes in the sequences were investigated and phylogeny was inferred. *Carnivore protoparvovirus* DNA was detected in 18 out of 96 dogs (18.8%) and 7 of 100 cats (7%). Phylogenetic analyses based on the full VP2 gene revealed that 9 sequenced strains clustered with different CPV clades (5 with 2c, 2 with 2a, 1 with 2b, and 1 with 2) and 1 strain with the FPV clade. All three CPV variants were detected in dog and cat populations with a predominance of CPV-2c strains (7 of 18, 38.9%) in dog samples, thus mirroring the circulation of this variant in African, European, and Asian countries. Deduced amino acid sequence alignment revealed the presence of the previously unreported unique mutations S542L, H543Q, Q549H, and N557T in the Egyptian CPV-2c strains.

## Introduction

Canine parvovirus (CPV) and feline panleukopenia virus (FPV) [species *Carnivore protoparvovirus 1* (CPPV 1), genus *Protoparvovirus*, family *Parvoviridae*] have been circulating globally in domestic dog and cat populations for several decades (1, 2). These viruses are highly pathogenic in their hosts, especially in young puppies and kittens, causing a severe enteric disease and requiring systematic vaccination for its prevention (3). Parvoviruses are small (diameter of 25 nm), non-enveloped viruses infecting vertebrates and insects. The virion consists of a spherical capsid, which is composed by three proteins (VP1, VP2, and VP3), with VP2 forming two-thirds of the capsid and being responsible for host range and immune response (4). The genome is a positive-sense, single-stranded DNA (4.5–5.5 kb), with complex hairpin-like structures at the 5′ and 3′ ends. The coding region of the genome contains two major expression cassettes, with open reading frames (ORFs) on the left-hand side giving rise to non-structural (NS) proteins (*ORF1*), whereas mRNA populations responsible for translating structural protein (viral proteins; VPs) are transcribed from the right-hand cassette (*ORF2*) (1, 2, 5).

Feline panleukopenia virus has been known since 1928 (6, 7) and is genetically and antigenically similar to CPV (8). CPV was first identified in the late 1970s when severe hemorrhagic gastroenteritis and myocarditis were reported in puppies (9). The virus was initially designated CPV-2 to distinguish it from the genetically unrelated CPV type 1 (currently known as *Carnivore bocaparvovirus 1*), but nowadays, CPV-2 generally refers to the original strain (3). It is speculated that CPV-2 has evolved from FPV after crossing the species barriers by acquiring a few amino acid mutations in the VP2 protein (10, 11). Shortly after its emergence, CPV-2 started evolving, thereby generating three antigenic variants, namely, CPV-2a, 2b, and 2c, which spread and substituted the original strain (12–16). CPV has shown a higher mutation rate than FPV, with the capsid protein gene mutating faster than the NS regions of the genome (17, 18). Consequently, the hypervariable VP2 protein remains the focus in CPV characterization. The numerous mutations reported over the years are postulated to provide advantages to the virus in the form of antigenic variation, capsid stability, and improved receptor-binding capacity, thus extending the host range and increasing the pathogenicity of new variants of the virus (13, 15, 19). The ability of these viruses to infect several wild carnivores further complicates their control as spillover infections occur from these animals to domestic pets and vice versa (20–22). CPV has also been shown to have gained the ability to infect domestic cats (23), highlighting the need for constant surveillance of these carnivores alongside the canine species.

The first report on CPV in Egypt dates back to 1982 (24). Since then, all three variants of the virus have been reported along with FPV (25–28). However, the presence of these viruses was assessed by serological tools or by molecular amplification of short fragments of the VP2 gene, providing limited genetic information on the circulation of CPPVs in Egypt. In the present paper, we report the characterization of CPPV strains from Egyptian domestic carnivores on the basis of the analysis of the full VP2 gene.

## Materials and methods

### Sample collection

A total of 196 blood samples previously collected from dogs (*n* = 96) and cats (*n* = 100) presented at the veterinary clinics for bacteriological surveillance from August to September 2019 were included in this study.

**Figure 1:**
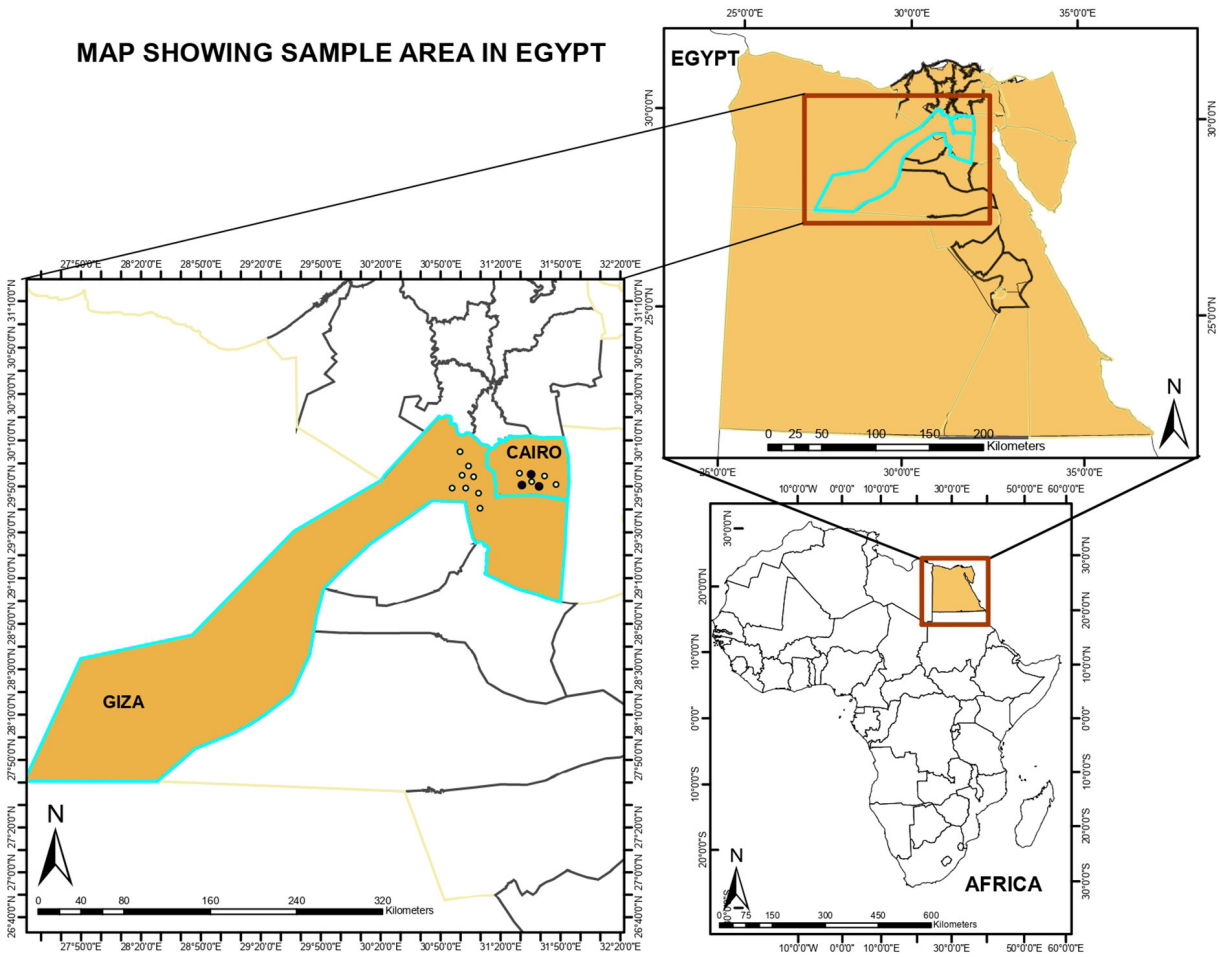
Shape files of study locations were obtained from the ArcGIS online map tools and imported for visualization into ArcGIS version 10.8.1 Redlands, CA: Environmental Systems Research Institute, Inc., 2020.

Samples were briefly stored at −20°C at the collection points and subsequently transported under a cold chain to the Infectious Diseases Unit of the Department of Veterinary Medicine, University of Bari for analysis. The samples were obtained from Cairo (*n* = 125) and Giza (*n* = 71), Egypt (Figure 1), and general information regarding the animals were collected.

### DNA extraction and screening for CPPV DNA

Viral DNA was extracted from the samples using the IndiSpin Pathogen Kit (Indical Bioscience GmbH, Leipzig, Germany), following the instructions of the manufacturer, and stored at −80°C until use. Quantification of extracted DNA was performed with the Fluorometric Qubit dsDNA High Sensitivity Assay Kit (Thermo Fisher Scientific, Waltham, MA, USA). The quality of the extracted DNA was compared by measuring the concentration and purity using a UV Spectrophotometer (NanoDrop™ 1000, Thermo Fisher Scientific). Testing of samples was done in three steps, the first of which involved the screening for the presence of CPV/FPV DNA using a real-time PCR (qPCR) assay based on TaqMan technology (29). This was followed by the characterization of positive samples by minor groove binder (MGB) probe-based qPCR assays to differentiate CPV types 2a/2b and 2b/2c and to discriminate between CPV and FPV (30) (Supplementary Table 1). Real-time PCR assays were performed using iTaq™ Universal Probes Supermix (Bio-Rad Laboratories SRL, Segrate, Italy). To evaluate the sensitivity of qPCR assays, 10-fold serial plasmid dilutions (10^8^-10^0^ DNA copy numbers per μl) containing the VP2 portion targeted by qPCR assays were used as templates. The repeatability was determined using four different DNA concentrations of CPPV tested. Concentrations of the DNA standard 10^2^, 10^3^, 10^5^, and 10^7^ DNA copy numbers per μl were tested and analyzed by qPCR. A total of 10 samples per reaction were tested to estimate the intra- and inter-assay coefficients. For intra-assay variability, each dilution was analyzed in triplicate. To evaluate the inter-assay precision of the assay, each dilution was analyzed in different runs performed by two different laboratory technicians on different days. The coefficient of variation (CV) was determined following the formula: CV = [SD (Ct-value)/overall mean (Ct-value)] × 100.

### Variable categorization and analyses

Data collated regarding clinical signs, age, and sampling location were sorted and inputted into Microsoft Excel (Microsoft Corporation, Redmond, WA, USA). Further, data were exported into SPSS (version 22; IBM Corp., Armonk, NY, USA), where descriptive and inferential statistical analyses were conducted. Age, clinical signs, and sampling location were categorized with corresponding cell values assigned in a “2 × 2” contingency matrix and the association between CPPV positivity and categorized variables was assessed by the chi-square test. *p* < 0.05 was considered statistically significant.

### Mapping

Shape files of study locations were obtained from the ArcGIS online map tools and imported for visualization into ArcGIS version 10.8.1 Redlands, CA: Environmental Systems Research Institute, Inc., 2020 (Figure 1).

### VP2 gene amplification and sequencing

VP2 gene was amplified in two overlapping fragments from samples that tested positive by qPCR, using primer sets previously described (17, 31) (Supplementary Table 1). Each 50 μl PCR reaction contained 5 μl of DNA extract, TaKaRa LA Taq ^TM^ Kit (Takara Bio Europe S.A.S. Saint-Germain-en-Laye, France), consisting of 24.5 μl of PCR grade water, 5 μl of 10× buffer, 5 μl of MgCl_2_ (25 mM), 1 μl of forward and reverse primers (50 μM), 8 μl of deoxynucleotide triphosphates (dNTPs) (2.5 mM), and 0.5 μl of Takara La Taq polymerase (5 U/μl). Initial denaturation was set at 94°C for 2 min, followed by 40 cycles of denaturation at 94°C for 1 min, 30 s annealing at 59°C for 1 min and extension at 68°C for 2 min, and a final extension at 68°C for 10 min. A DNA extract of an FPV vaccine (Vanguard^®^ Feline RCP, Zoetis) and nuclease-free water were included as positive control and blank, respectively. The PCR products were electrophoresed in a 1.5% agarose gel at 80 V for 40 min and the amplification bands were visualized on a Gel Doc™ EZ (Bio-Rad Laboratories SRL, Segrate, Italy), using Image Lab^TM^ software.

### Sequencing and sequence analysis

Purification of PCR products was performed by QIAquick PCR Purification Kit (Qiagen GmbH, Hilden, Germany), followed by nucleotide (nt) sequencing in both directions by the Sanger method using BigDye 3.1 Ready Reaction Mix (Applied Biosystems), according to the instructions of the manufacturer. Generated reads were edited, and contigs were assembled using Geneious Prime version 2021.1 (Biomatters, Auckland, New Zealand). Related sequences were explored using web-based tools Basic Local Alignment Search Tool (BLAST; https://blast.ncbi.nlm.nih.gov/Blast.cgi?PAGE_TYPE=BlastSearch) and FAST-All (FASTA; https://www.ebi.ac.uk/Tools/sss/fasta/nucleotide.html). The obtained sequences were aligned with reference CPPV sequences retrieved from the GenBank database by the multiple alignment using fast Fourier transform (MAFFT) algorithm (32).

### Sequence submission

Nucleotide sequences of strains EGY/2019/39-122, EGY/2019/39-134, EGY/2019/39-161, EGY/2019/39-167, EGY/2019/39-168, EGY/2019/39-178, EGY/2019/39-200, EGY/2019/39-517, EGY/2019/39-549, and EGY/2019/39-566 used for phylogeny were deposited in the GenBank under the accession nos. OM937907, OM937916.

### Phylogenetic analysis

The most appropriate model of evolution for phylogenetic analysis on the full VP2 gene of CPPV strains was evaluated using a jModelTest software (http://evomics.org/resources/software/molecular-evolution-software/modeltest/). The identified program settings for all partitions, under the Bayesian Information Criteria, included 5-character states (general time-reversible model), a proportion of invariant sites, and a discrete gamma distribution (6 categories) of rate variation across sites. Phylogenetic analyses were conducted using MrBayes 4 chains run for >1 million generations (33, 34) and a bootstrap analysis with 1,000 pseudoreplicated datasets.

Phylogenetic analyses using other evolutionary models (maximum likelihood and neighbor joining) were performed to compare the topology of the phylogenetic trees. Similar topologies with slight differences in bootstrap values at the nodes of the tree were observed. Accordingly, the Bayesian tree was retained.

## Results

### CPV diagnosis and statistical analysis

In the screening performed using a qPCR assay based on TaqMan technology, CPPV DNA was detected in a total of 25 animals consisting of 18 of 96 dogs (18.8%) and 7 of 100 cats (7%) (Table 1). The detection limit of the CPPV qPCR was 10^1^ DNA copy numbers per μl. The qPCR assay expressed a high repeatability with CV within runs (intra-assay variability) and between runs (inter-assay variability) that ranged from 0.73 to 1.69% and 0.97 to 2.18%, respectively. Overall, the viral load of CPPV in samples ranged from 1.6 × 10^1^ to 1.1 × 10^5^ DNA copy numbers per μl (mean 7.3 × 10^3^ DNA copy numbers per μl, median 2.1 × 10^3^ DNA copy numbers per ml). In the MGB probe-based qPCR assays, the most frequently detected variant in dogs was CPV-2c (*n* = 7/18, 38.9%), followed by CPV-2a (*n* = 2/18, 11.1%), CPV-2b (1/18, 5.6%), and CPV-2 1/18 dog (5.6%). Due to lower viral load (mean 2.1 × 10^2^ DNA copy numbers per μl, median 1.1 × 10^2^ DNA copy numbers per μl), 7 of 18 CPV-positive samples (38.9%) identified in dogs could not be characterized by MGB probe-based qPCR assays. Out of a total of 7 cats, FPV and CPV-2a were identified in 3 (42.9%) and 2 (28.5%) cats, respectively. One cat (14.3%) tested positive for CPV-2b, while in another cat (14.3%), the concurrent presence of CPV-2a and−2c DNA was observed.

**Table 1:** Inferential statistics testing the association between socio-demographics, clinical signs, and disease outcome in the sampled dogs and cats.

				**PCR**			
**Animal species**	**Agent**	**Variable**	**Category**	**Positive N (%)**	**Negative N (%)**	**df**	***p*** **-value**
Dog	CPV	Location	Cairo	1 (5.6)	24 (30.8)	1	0.021
			Giza	17 (94.4)	54 (69.2)		
			Total	18 (100.0)	78 (100.0)		
		Clinical signs	Anemia	2 (11.1)	32 (41.0)	2	0.03
			Fever	12 (66.7)	28 (35.9)		
			Low weight	4 (22.2)	18 (23.1)		
			Total	18 (100.0)	78 (100.0)		
		Age	≤ 6 months	17 (94.4)	75 (96.2)	1	0.571
			>6 months	1 (5.6)	3 (3.8)		
			Total	18 (100.0)	78 (100.0)		
Cat	FPV/CPV	Clinical signs	Anemia	2 (28.6)	11 (11.8)	2	0.381
			Fever	4 (57.1)	73 (78.5)		
			Low weight	1 (14.3)	9 (9.7)		
			Total	7 (100.0)	93 (100.0)		
		Age	≤ 6 months	7 (100.0)	90 (96.8)	1	0.803
			>6 months	0 (0.0)	3 (3.2)		
			Total	7 (100.0)	100 (100.0)		


The total mean age in weeks of the 100 cats and 96 dogs tested was 13.54 ± 0.5 and 13.69 ± 4.92, respectively. While all the cat samples tested were collected only from Cairo, a significantly higher number of CPV-positive dogs were reported for Giza in comparison to Cairo (*p* = 0.021). In addition, a significant association was found between the presence of CPPV-1 DNA and clinical signs, with fever identified as the most observed clinical sign in disease outcomes (*p* = 0.03). Similarly, fever was more consistently associated with feline FPV and CPV than anemia or low weight, although this association was not statistically significant (*p* > 0.05). All positive cat cases occurred in kittens of 6 months of age or under (*n* = 7), although without statistical significance (Table 1).

### VP2 sequence and phylogenetic analyses

Out of 25 CPPV strains detected by qPCR, 10 full VP2 sequences (7 from dog samples and 3 from cat samples) were successfully amplified by conventional PCR and sequenced. BLAST and FASTA analyses revealed a high nt identity with other reference sequences from the GenBank database (99.6–100%) while identity within the sequences from this study was 97.8–100%. By sequence comparison of amino acid (aa) residues of FPV strains identified in the present study with cognate reference sequences, no aa substitutions were observed (Table 2). Conversely, eleven non-synonymous mutations were found in the CPV sequences from this study, compared to cognate reference sequences used in the phylogenetic tree (Table 2). CPV-2 strain EGY/2019/39-122 (OM937907) displayed three aa substitutions at positions 219 (L → V), 386 (Q → K), and 418 (I → T), while CPV2a strain EGY/2019/39-200 (OM937913) exhibited two aa substitutions at positions 13 (P → S) and 440 (T → A). CPV-2c strain EGY/2019/39-167 (OM937910) displayed two aa substitutions at positions 549 (Q → H) and 577 (N → T). Unique aa substitutions were observed in the CPV-2b strain EGY/2019/39-517 (OM937914) at position 440 (T → A), in the CPV-2c strain EGY/2019/dog/39-134 (OM937908) at position 542 (S → L), and in the CPV-2c strain EGY/2019/39-168 at position 543 (H → Q). All the CPV variants identified in Egyptian samples displayed aa substitution S → A at position 297.

**Table 2:** Summary table of nucleotide and amino acid substitutions in the VP2 region of carnivore protoparvoviruses (canine parvovirus 2, CPV-2 and feline panleukopenia virus, FPV) detected in blood samples of dogs and cats in Egypt as compared to reference strains used for the phylogeny (Figure 2).

**aa position**	**13**	**219**	**297**	**386**	**418**	**426**	**440**	**542**	**543**	**549**	**557**
Nucleotide position	(37–39)	(655–657)	(889–891)	(1,156–1,158)	(1,252–1,254)	(1,276–1,278)	(1,318–1,320)	(1,624–1,626)	(1,629–1,320)	(1,645–1,647)	(1,669–1,671)
CPV-2/EGY/2019/dog/39-122-OM937907	P (CCT)	V (GTA)	S (TCT)	K (AAA)	T (ACT)	N (AAT)	T (ACA)	S (TCT)	H (CAT)	Q (CAA)	N (AAC)
CPV-2a/EGY/2019/cat/39-549-OM937915	P (CCT)	I (ATA)	A (GCT)	Q (CAA)	I (ATT)	N (AAT)	A (GCA)	S (TCT)	H (CAT)	Q (CAA)	N (AAC)
CPV-2a/EGY/2019/dog/39-200-OM937913	S (TCT)	I (ATA)	A (GCT)	Q (CAA)	I (ATT)	N (AAT)	A (GCA)	S (TCT)	H (CAT)	Q (CAA)	N (AAC)
CPV-2b/EGY/2019/cat/39-517-OM937914	P (CCT)	I (ATA)	A (GCT)	Q (CAA)	I (ATT)	D (GAT)	A (GCA)	S (TCT)	H (CAT)	Q (CAA)	N (AAC)
CPV-2c/ EGY/2019/dog/39-134-OM937908	P (CCT)	I (ATA)	A (GCT)	Q (CAA)	I (ATT)	E (GAA)	T (ACA)	L (CTC)	H (CAT)	Q (CAA)	N (AAC)
CPV-2c/EGY/2019/dog/39-168-OM937911	P (CCT)	I (ATA)	A (GCT)	Q (CAA)	I (ATT)	E (GAA)	T (ACA)	S (TCT)	Q (CAA)	Q (CAA)	N (AAC)
CPV-2c/EGY/2019/dog/39-161-OM937909	P (CCT)	I (ATA)	A (GCT)	Q (CAA)	I (ATT)	E (GAA)	T (ACA)	S (TCT)	H (CAT)	Q (CAA)	N (AAC)
CPV-2c/EGY/2019/dog/39-167-OM937910	P (CCT)	I (ATA)	A (GCT)	Q (CAA)	I (ATT)	E (GAA)	T (ACA)	S (TCT)	H (CAT)	H (CAC)	T (ACC)
CPV-2c/EGY/2019/dog/39-178-OM937912	P (CCT)	I (ATA)	A (GCT)	Q (CAA)	I (ATT)	E (GAA)	T (ACA)	S (TCT)	H (CAT)	Q (CAA)	N (AAC)
FPV/EGY/2019/cat/39-566- OM937916	P (CCT)	I (ATA)	S (TCT)	Q (CAA)	I (ATT)	N (AAT)	T (ACA)	S (TCT)	H (TAC)	Q (CAA)	N (AAC)
CPV-2/CHN/2019/dog/CC-33-MN810900	P (CCT)	V (GTA)	S (TCT)	K (AAA)	T (ACT)	N (AAT)	T (ACA)	S (TCT)	H (CAT)	Q (CAA)	N (AAC)
CPV-2/IND/2011/dog/vac4-JN625222	P (CCT)	V (GTA)	S (TCT)	K (AAA)	I (ATT)	N (AAT)	T (ACA)	S (TCT)	H (CAT)	Q (CAA)	N (AAC)
CPV-2/ITA/2005/dog/388.05-3-FJ222824	P (CCT)	V (GTA)	S (TCT)	K (AAA)	I (ATT)	N (AAT)	T (ACA)	S (TCT)	H (CAT)	Q (CAA)	N (AAC)
CPV-2/USA/1979/dog/5.us.79-EU659116	P (CCT)	I (ATA)	S (TCT)	Q (CAA)	I (ATT)	N (AAT)	T (ACA)	S (TCT)	H (CAT)	Q (CAA)	N (AAC)
CPV-2/USA/1990/dog/790312-M38245	P (CCT)	I (ATA)	S (TCT)	Q (CAA)	I (ATT)	N (AAT)	T (ACA)	S (TCT)	H (CAT)	Q (CAA)	N (AAC)
CPV-2/USA/1988/dog/N-M19296	P (CCT)	I (ATA)	S (TCT)	Q (CAA)	I (ATT)	N (AAT)	T (ACA)	S (TCT)	H (CAT)	Q (CAA)	N (AAC)
CPV-2a new/CHN/2018/dog/AHmas16-MT648208	P (CCT)	I (ATA)	A (GCT)	Q (CAA)	I (ATT)	N (AAT)	A (GCA)	S (TCT)	H (CAT)	Q (CAA)	N (AAC)
CPV-2a/CHN/2015/dog/BJL1-MH106698	P (CCT)	I (ATA)	A (GCT)	Q (CAA)	I (ATT)	N (AAT)	A (GCA)	S (TCT)	H (CAT)	Q (CAA)	N (AAC)
CPV-2a/IRN/2020/dog/22-MW653250	P (CCT)	I (ATA)	A (GCT)	Q (CAA)	I (ATT)	N (AAT)	A (GCA)	S (TCT)	H (CAT)	Q (CAA)	N (AAC)
CPV-2a new/CHN/2016/dog/10-MF805798	P (CCT)	I (ATA)	A (GCT)	Q (CAA)	I (ATT)	N (AAT)	A (GCA)	S (TCT)	H (CAT)	Q (CAA)	N (AAC)
CPV-2a new/IND/2018/dog/TN-MH545963	P (CCT)	I (ATA)	A (GCT)	Q (CAA)	I (ATT)	N (AAT)	A (GCA)	S (TCT)	H (CAT)	Q (CAA)	N (AAC)
CPV-2a new/IND/2020/dog/ABT03-MT441832	P (CCT)	I (ATA)	A (GCT)	Q (CAA)	I (ATT)	N (AAT)	A (GCA)	S (TCT)	H (CAT)	Q (CAA)	N (AAC)
CPV-2a/URY/2011/dog/recUY364-KM457139	P (CCT)	I (ATA)	A (GCT)	Q (CAA)	I (ATT)	N (AAT)	A (GCA)	S (TCT)	H (CAT)	Q (CAA)	N (AAC)
CPV-2b/THA/2015/dog/VT123-KP715712	P (CCT)	I (ATA)	A (GCT)	Q (CAA)	I (ATT)	D (GAT)	T (ACA)	S (TCT)	H (CAT)	Q (CAA)	N (AAC)
CPV-2b/TUR/2020/dog/I1-MW539053	P (CCT)	I (ATA)	A (GCT)	Q (CAA)	I (ATT)	D (GAT)	A (GCA)	S (TCT)	H (CAT)	Q (CAA)	N (AAC)
CPV-2c/CHN/2020/dog/XA-1-MZ506743	P (CCT)	I (ATA)	A (GCT)	Q (CAA)	I (ATT)	E (GAA)	T (ACA)	S (TCT)	H (CAT)	Q (CAA)	N (AAC)
CPV-2c/CHN/2020/dog/ZJHN-136-MW017617	P (CCT)	I (ATA)	A (GCT)	Q (CAA)	I (ATT)	E (GAA)	T (ACA)	S (TCT)	H (CAT)	Q (CAA)	N (AAC)
CPV-2c/CHN/2017/dog/SH1516-MG013488	P (CCT)	I (ATA)	A (GCT)	Q (CAA)	I (ATT)	E (GAA)	T (ACA)	S (TCT)	H (CAT)	Q (CAA)	N (AAC)
CPV-2c/CHN/2019/dog/AHhf27-MT648203	P (CCT)	I (ATA)	A (GCT)	Q (CAA)	I (ATT)	E (GAA)	T (ACA)	S (TCT)	H (CAT)	Q (CAA)	N (AAC)
CPV-2c/ITA/2017/dog/IZSSI_2743_17-MF510157	P (CCT)	I (ATA)	A (GCT)	Q (CAA)	I (ATT)	Glu (GAA)	T (ACA)	S (TCT)	H (CAT)	Q (CAA)	N (AAC)
CPV-2c/NGA/2018/dog/IZSSI_PA1464-MT840293	P (CCT)	I (ATA)	A (GCT)	Q (CAA)	I (ATT)	E (GAA)	T (ACA)	S (TCT)	H (CAT)	Q (CAA)	N (AAC)
CPV-2c/ROU/2019/dog/161-MW659473	P (CCT)	I (ATA)	A (GCT)	Q (CAA)	I (ATT)	E (GAA)	T (ACA)	S (TCT)	H (CAT)	Q (CAA)	N (AAC)
CPV-2c/THA/2016/dog/CU24-MH711894	P (CCT)	I (ATA)	A (GCT)	Q (CAA)	I (ATT)	E (GAA)	T (ACA)	S (TCT)	H (CAT)	Q (CAA)	N (AAC)
CPV-2c/VNM/2013/dog/HCM-7-LC214969	P (CCT)	I (ATA)	A (GCT)	Q (CAA)	I (ATT)	E (GAA)	T (ACA)	S (TCT)	H (CAT)	Q (CAA)	N (AAC)
CPV-2c/CHN/2016/dog/YZ1-MF001435	P (CCT)	I (ATA)	A (GCT)	Q (CAA)	I (ATT)	E (GAA)	T (ACA)	S (TCT)	H (CAT)	Q (CAA)	N (AAC)
CPV-2c/NGA/2018/dog/IZSSI PA1464/19 idYV2-MK895486	P (CCT)	I (ATA)	A (GCT)	Q (CAA)	I (ATT)	E (GAA)	T (ACA)	S (TCT)	H (CAT)	Q (CAA)	N (AAC)
FPV/THA/2020/cat/TRC-B88-MW589472	P (CCT)	I (ATA)	S (TCT)	Q (CAA)	I (ATT)	N (AAT)	T (ACA)	S (TCT)	H (TAC)	Q (CAA)	N (AAC)
FPV/THA/2018/cat/18R217C-MN127779	P (CCT)	I (ATA)	S (TCT)	Q (CAA)	I (ATT)	N (AAT)	T (ACA)	S (TCT)	H (TAC)	Q (CAA)	N (AAC)
FPV/CHN/1999/tiger/G-MG764510	P (CCT)	I (ATA)	S (TCT)	Q (CAA)	I (ATT)	N (AAT)	T (ACA)	S (TCT)	H (TAC)	Q (CAA)	N (AAC)
FPV/IND/2018/cat/TN-MH559110	P (CCT)	I (ATA)	S (TCT)	Q (CAA)	I (ATT)	N (AAT)	T (ACA)	S (TCT)	H (TAC)	Q (CAA)	N (AAC)
FPV/SKR/2017/cat/Gigucheon-MN400978	P (CCT)	I (ATA)	S (TCT)	Q (CAA)	I (ATT)	N (AAT)	T (ACA)	S (TCT)	H (TAC)	Q (CAA)	N (AAC)
FPV/ITA/2003/cat/189.03-EU498686	P (CCT)	I (ATA)	S (TCT)	Q (CAA)	I (ATT)	N (AAT)	T (ACA)	S (TCT)	H (TAC)	Q (CAA)	N (AAC)
FPV/ITA/2017/cat/880007-MW847187	P (CCT)	I (ATA)	S (TCT)	Q (CAA)	I (ATT)	N (AAT)	T (ACA)	S (TCT)	Q (GAC)	Q (CAA)	N (AAC)
FPV/ITA/2015/cat/IZSSI_3201_1_15-KX434461	P (CCT)	I (ATA)	S (TCT)	Q (CAA)	I (ATT)	N (AAT)	T (ACA)	S (TCT)	Q (TAC)	Q (CAA)	N (AAC)
FPV/USA/1964/cat/4.us_64-EU659112	P (CCT)	I (ATA)	S (TCT)	Q (CAA)	I (ATT)	N (AAT)	T (ACA)	S (TCT)	Q (TAC)	Q (CAA)	N (AAC)
FPV/AUS/1970/cat/193-X55115	P (CCT)	I (ATA)	S (TCT)	Q (CAA)	I (ATT)	N (AAT)	T (ACA)	S (TCT)	Q (TAC)	Q (CAA)	N (AAC)
FPV/CAN/2017/american pine marten/MAHG-3-MN862745	P (CCT)	I (ATA)	S (TCT)	Q (CAA)	I (ATT)	N (AAT)	T (ACA)	S (TCT)	Q (TAC)	Q (CAA)	N (AAC)

**Figure 2:**
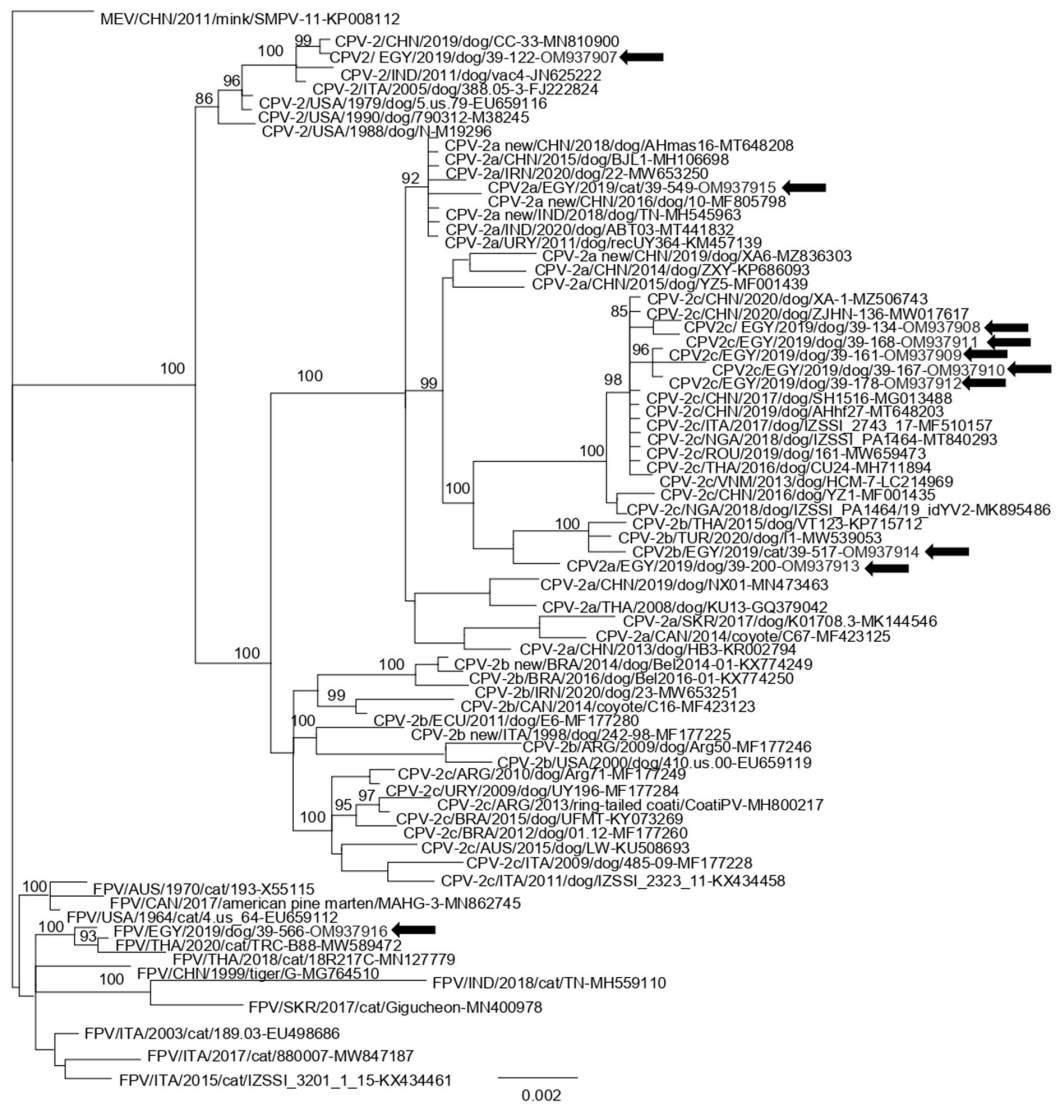
Bayesian open reading frame (ORF)2-based phylogenetic tree of Carnivore protoparvovirus 1. The tree was elaborated using a 1,755 nt long alignment of the ORF2 sequence of the Egyptian canine parvovirus (CPV) and feline panleukopenia virus (FPV) strains identified in this study and the cognate sequences of Carnivore protoparvovirus 1 strains retrieved from the GenBank database. The posterior output of the tree was derived using a general time-reversible model, a proportion of invariable sites, a gamma distribution of rate variation across sites, and a subsampling frequency of 1,000. Posterior probability values >95% are indicated at the tree nodes. The black arrows indicate the Egyptian strains generated in this study. The scale bar indicates the number of nt substitutions per site.

Phylogenetic analysis revealed that 5 strains (EGY/2021/39-134, EGY/2021/39-168, EGY/2021/39-161, EGY/2021/39-167, and EGY/2021/39-178) segregated into the CPV-2c clade together with European, Asian, and Nigerian strains. Strain EGY/2021/39-517 clustered with Turkish and Thai CPV-2b strain while with strain EGY/2021/39-200, characterized as CPV-2a, was immediately basal to the clade. Strain EGY/2021/39-549 segregated into CPV-2a clade together with other strains identified in China, Middle East, and Uruguay. Strain EGY/2021/39-122 clustered with CPV-2 strains retrieved from the USA, Italy, China, and India, while strain EGY/2021/39-566 segregated with FPV strains identified in Thailand (Figure 2).

## Discussion

Carnivore protoparvoviruses have maintained their status as a major cause of mortality, especially in juvenile dogs and cats globally, despite decades of vaccine use. Mutations in the hypervariable capsid gene VP2 are of importance as they are known to influence virus/receptor binding, thereby playing key roles as determinants of host range and antigenicity (10, 35–38). These mutant viruses also tend to acquire evolutive advantages, thriving, spreading, and replacing existing variants, thus, driving the dynamic epidemiology of CPV across countries. The use of blood samples in this study rather than feces cannot account for the overall low detection rate, as in infected animals, viremia is longer lasting than fecal shedding (39), and CPPVs have been frequently detected also in the blood of healthy dogs and cats (40). Previous studies in Egypt based their selection criteria on the development of diarrhea, followed by positivity to rapid tests, resulting in the prevalence of 84% and higher (28, 41).

This study confirms the circulation of all three CPV variants in domestic dog and cat populations from Egypt. CPV is known to infect cats and has been suggested to contribute to genetic diversity of CPPV as a consequence of infecting both dogs and cats (23, 42). CPV infections in cats are usually mild (42) but clinical cases resembling to feline panleukopenia have been reported (23). Cats have been also found to shed CPV without clinical signs (43, 44), serving as reservoir hosts. The present study, however, cannot rule out the role of other blood-borne pathogens in the induction of fever in the animals tested. A lack of vaccination and of history of gastroenteric disease in the animals limits the interpretation of the clinical significance of this study.

A significantly higher number of CPV-positive dogs were reported for Giza (17/71, 23.9%) as compared to Cairo (1/25, 0.04%) (Table 1). While Cairo is more densely human-populated, Giza is home to the popular pyramids and attracts a huge number of tourists from around the world throughout the year. The intensive movements of people (and their pets) from diverse origins might favor the introduction of new pathogens into the region, possibly through fomites. In addition, the complexity of the host immunity response against the CPPV vaccine cannot rule out the possibility of a vaccinated animal getting infected following vaccination or shedding wild-type virus without clinical signs.

A total of 10 CPPV strains have been characterized in this study. The CPV-2 strain EGY/2019/39-122 was 100% identical to isolate CC-33 (MN810900) identified from a dog in China in 2019. CPV-2 has been sporadically detected in other studies, usually as a consequence of recent vaccination (45, 46), since the original strain is no longer circulating in the field, but it is still contained in a number of vaccines (3). The CPV-2 strain displayed the presence of Val-219 and Lys-386 as also observed in the VP2 of the Nobivac^®^ vaccine (C3) (MG264079) (47), and Thr-418 has been previously described in CPPV strains from domestic and wild carnivores (20, 48).

CPV-2a strain EGY/2019/dog/39-200 displayed the mutation S13P consistently reported in Italy in the last decades (48–50). Both CPV-2a strain EGY/2019/dog/39-200 (OM937913) and CPV-2b strain EGY/2019/cat/39-517 (OM937914) displayed the mutation T440A and were closely related to the isolates from Turkey and Thailand (51, 52). The T440A mutation was also prevalent in the CPV-2a strains previously identified in Egypt (53). CPV variants displaying VP2 changes F267Y, Y342I, and T440A are considered immune escape mutants, which are likely emerged due to vaccine pressure, with the role of the 267 mutation still unclear although it is an unexposed residue (37, 46). Residues 324 and 440 are located next to the spike residues 423 and 427, respectively (37).

CPV-2c was the predominant variant circulating in domestic dogs in Egypt, in contrast to earlier reports that accounted for a limited circulation of CPV-2c in this country (28, 53). The CPV-2c mutant detected has been also reportedly spreading in Europe, Asia, and Africa (54–63). This variant was recently detected in Nigeria (64) and is widespread in this country (63, 64). Considering the rapid spreading of this CPV-2c mutant, and Egypt being a touristic country, a predominance of this variant is expected in the next few years.

Amino acid substitutions observed in the CPV2c strains EGY/2019/39-21-134 (S542L), EGY/2019/39-167 (Q549H, N557T), and EGY/2019/39-168 (H543Q) were unique and have not been previously reported.

All CPV variants identified in this study displayed a VP2 with Ala-297, a recent widespread mutation (S → A) due to host adaptation (31, 65–70). The residue 297 is under strong positive selection pressure (68) and mutants displaying such a change have been considered a subvariant of CPV-2a/2b (46).

The FPV sequence from this study was 99.7% identical to FPV TRC-B88/TH/2020, which had been detected in the brain of a cat in Thailand. Overall, the FPV genome has a lower mutation rate than CPV (18), hence the observation of nt changes with no effect on the VP2 sequence is more frequent in the former.

A more extensive epidemiological surveillance is needed in domestic carnivores from Egypt and other African countries in order to better understand the evolution and variability of CPPV in geographic areas where the epidemiological data of these viruses are still scarce.

## Data availability statement

The datasets presented in this study can be found in online repositories. The names of the repository/repositories and accession number(s) can be found in the article/supplementary material.

## Ethics statement

The animal study was reviewed and approved by Medical research Ethics Committee of the National Research Centre, Cairo, Egypt (approval number 6211022021). Written informed consent was obtained from the owners for the participation of their animals to this study.

## Author contributions

ND, CB, and GL designed the experiment. LN, CD, EO, AZ, and PC carried out the experiment. LN, GL, and PC wrote the manuscript. LN, GL, FE, and GG participated in the data analysis. ND and CB reviewed the manuscript. All authors read and approved the manuscript before submission.

## Funding

This study was supported by grants from the Italian Ministry of Health: Ricerca Corrente 2019 NGS e diagnostica molecolare in Sanità Animale: Fast D2, recipient ND.

## Conflict of interest

The authors declare that the research was conducted in the absence of any commercial or financial relationships that could be construed as a potential conflict of interest.

## Publisher's note

All claims expressed in this article are solely those of the authors and do not necessarily represent those of their affiliated organizations, or those of the publisher, the editors and the reviewers. Any product that may be evaluated in this article, or claim that may be made by its manufacturer, is not guaranteed or endorsed by the publisher.
