# Supplementary material for: Molecular characterization of carnivore protoparvovirus 1 circulating in domestic carnivores in Egypt
Source: Front Vet Sci. 2022 Jul 22;9:932247. doi: 10.3389/fvets.2022.932247 (PMC9354892; doi:10.3389/fvets.2022.932247)
Supplement: Supplementary file 1 [file Table_1.DOCX]

|  | Primer/probe | Sequence 5’ to 3’ | Target | Amplicon size | Reference | |
| --- | --- | --- | --- | --- | --- | --- |
| *Carnivore protoparvovirus-1* TaqMan assay | CPV-For | AAACAGGAATTAACTATACTAATATATTTA | FPLV/CPV | 93 bp | | (29) |
|  | CPV-Rev | AAATTTGACCATTTGGATAAACT |  |  |  |  |
|  | CPV-Pb | FAM –TGGTCCTTTAACTGCATTAAATAATGTACC - TAMRA |  |  |  |  |
| FPV/CPV assay | FPV/CPV-For | ACAAGATAAAAGACGTGGTGTAACTCAA | FPLV/CPV | 83 bp | | (17) |
|  | FPV/CPV-Rev | CAACCTCAGCTGGTCTCATAATAGT |  |  |  |  |
|  | FPV-Pb | VIC – ATGGGAAATACAGACTATAT - MGB | FPLV |  |  |  |
|  | CPV-Pb | FAM – ATGGGAAATACAAACTATAT - MGB | CPV |  |  |  |
| CPV-2a/2b assay | CPVa/b-For | AGGAAGATATCCAGAAGGAGATTGGA | All CPV |  | | (30) |
|  | CPVa/b-Rev | CCAATTGGATCTGTTGGTAGCAATACA | All CPV | 93 bp | |  |
|  | CPVa-Pb | VIC – CTTCCTGTAACAAATGATA - MGB | CPV-2a |  | |  |
|  | CPVb1-Pb | FAM – CTTCCTGTAACAGATGATA - MGB | CPV-2b |  | |  |
| CPV-2b/2c assay | CPVb/c-For | GAAGATATCCAGAAGGAGATTGGATTCA | All CPV |  | | (30) |
|  | CPVb/c-Rev | ATGCAGTTAAAGGACCATAAGTATTAAATATATTAGTATAGTTAATTC | All CPV | 150 bp | |  |
|  | CPVb2-Pb | FAM – CCTGTAACAGATGATAAT - MGB | CPV-2b |  | |  |
|  | CPVc-Pb | VIC – CCTGTAACAGAAGATAAT - MGB | CPV-2c |  | |  |
|  | CPV-2679F | CCAGATCATCCATCAACATCA | 2,649 -> 2,669 | 747 bp | |  |
|  | CPV-3511R | TGAACATCATCTGGATCTGTACC | 3,505 -> 3,483 |  | |  |
| PCR/Sequencing | F1 | AGATAGTAATAATACTATGCCATTT | 3,311 -> 3,335 | 1375 bp | | (31) |
|  | R1 | CCTATATCAAATACAAGTACAATA | 4,685 -> 4,662 |  |  |  |

**Table 1**: Primers used in real-time and conventional PCR in this study

FPV:feline panleukopenia virus; CPV:canine parvovirus
